# Highlights from the 1st Latin American meeting on metronomic chemotherapy and drug repositioning in oncology, 27–28 May, 2016, Rosario, Argentina

**DOI:** 10.3332/ecancer.2016.672

**Published:** 2016-09-06

**Authors:** Adriana Rosé, Nicolas André, Viviana R. Rozados, Leandro E Mainetti, Mauricio Menacho Márquez, María José Rico, Paula Schaiquevich, Milena Villarroel, Lauro Gregianin, Jaume Mora Graupera, Wendy Gómez García, Sidnei Epelman, Carlos Alasino, Daniel Alonso, Guillermo Chantada, O Graciela Scharovsky

**Affiliations:** 1 Hospital de Pediatría ‘JP Garrahan’, Combate de los Pozos 1800, C 1245 AAM, CABA Argentina; 2 Inserm UMR_S 911, Centre de Recherche en Oncologie Biologique et Oncopharmacologie, 27 Boulevard Jean Moulin, Faculté de Pharmacie, Aix-Marseille Université; Service d’Hématologie & Oncologie Pédiatrique, AP‑HM, 13005 Marseille, France; 3 Instituto de Genética Experimental, Facultad de Ciencias Médicas, Santa 3100, 2000 Rosario, Argentina; 4 Unidad de Farmacocinética Clínica, Hospital de Pediatría ‘JP Garrahan’, Combate de los Pozos 1800, C 1245 AAM, CABA Argentina; 5 Av Antonio Varas 360, Santiago, Providencia, Región Metropolitana, Chile; 6 Hospital de Clínicas de Porto Alegre, Serviço de Oncologia Pediátrica, Rua Ramiro Barcelos, 2350, Petrópolis, Porto Alegre, RS 90670150, Brazil; 7 Department of Paediatric Haemato-Oncology, Hospital Sant Joan de Déu, Passeig de Sant Joan de Déu, 2, 08950 Esplugues de Llobregat, Barcelona, Spain; 8 Hospital Infantil Dr Robert Reid Cabral, Servicio de Hem-Oncología HIRRC, Ave Abraham Lincoln 2, Casi Esq Ave, Independencia, Santo Domingo, Dominican Republic; 9 Paediatric Oncology Department, Santa Marcelina Hospital, R Rio Negro, 48, Itaquaquecetuba, São Paulo, SP 08599-280, Brazil; 10 Instituto de Oncología de Rosario, Córdoba 2457, S2000KZE Rosario, Argentina; 11 Laboratorio de Oncología Molecular, Universidad Nacional de Quilmes, Roque Sáenz Peña 352, B1876BXD Bernal, Buenos Aires, Argentina; 12 Instituto de Investigaciones, Hospital de Pediatría ‘JP Garrahan’, Combate de los Pozos 1800, C 1245 AAM, CABA Argentina

**Keywords:** cancer, metronomic chemotherapy, drug repositioning, pre-clinic, paediatric, adult

## Abstract

Following previous metronomic meetings in Marseille (2011), Milano (2014), and Mumbai (2016), the first Latin American metronomic meeting was held in the School of Medical Sciences, National University of Rosario, Rosario, Argentina on 27 and 28 of May, 2016. For the first time, clinicians and researchers with experience in the field of metronomics, coming from different countries in Latin America, had the opportunity of presenting and discussing their work. The talks were organised in three main sessions related to experience in the pre-clinical, and clinical (paediatric and adult) areas. The different presentations demonstrated that the fields of metronomic chemotherapy and repurposing drugs in oncology, known as metronomics, constitute a branch of cancer therapy in permanent evolution, which have strong groups working in Latin

America, both in the preclinical and the clinical settings including large, adequately designed randomised studies. It was shown that metronomics offers treatments, which, whether they are combined or not with the standard therapeutic approaches, are not only effective but also minimally toxic, with the consequent improvement of the patient’s quality of life, and inexpensive, a feature very important in low resource clinical settings. The potential use of metronomic chemotherapy was proposed as a cost/effective treatment in low-/middle-income countries, for adjuvant therapy in selected tumours. The fundamental role of the governmental agencies and non-governmental alliances, as the Metronomic Global Health Initiative, in supporting this research with public interest was underlined.

## Introduction

Following previous metronomic meetings in Marseille [1], Milano [2], and Mumbai (2016), the first Latin American metronomic meeting was held at the School of Medical Sciences, National University of Rosario, Rosario, Argentina on 27 and 28 of May, 2016. The organiser of the conference, Dr O Graciela Scharovsky from the Institute of Experimental Genetics of the School of Medical Sciences, gave a warm welcome to both speakers and attendants. She began by defining metronomic chemotherapy (MCT) and mentioning the first meaningful contributions to this novel therapeutic modality from Judah Folkman’s [3] and Robert Kerbel’s [4] laboratories. Those publications were followed by many others coming from different laboratories, including her own, which contributed to increasing the knowledge about mechanisms of action of metronomic chemotherapy and its translation to the clinic. Those results contributed to support changing the established paradigm in therapeutic oncology that ‘more is better’ by a new one ‘less is more, if administered chronically’. Also, in drug repositioning (DR), a new and feasible approach for cancer therapy, which has begun to be systematically studied since the ReDO project [5] was launched, a paradigm change is proposed, going from the previous ‘one drug, one target, one illness’ to the new one ‘one drug, several targets, several illnesses’ MCT and DR found a common pathway named ‘metronomics’ [6]. That is, by now, the route travelled by many scientists that have embraced this new therapeutic philosophy. Dr Scharovsky was positive that utilising metronomics, together with therapeutic weapons already in use, will enable us to make a positive difference in the treatment of cancer [7]. ‘It is our task to achieve the goal of MCT and DR eventually joining the “official” set of weapons that we employ to defeat cancer’.

## Opening lecture

Nicolas André, from Faculté de Médecine; Service d’Hématologie & Oncologie Pédiatrique, AP-HM, Metronomics Global Health Initiative, Marseille, France, gave the main lecture. He built a timeline for the progress in metronomics, beginning with Metronomics 1.0 section which started in 2000. MCT was originally developed to overcome drug resistance by shifting the therapeutic target from tumour cells to tumour vasculature. In the past decade, several pilot phase-II and phase-III clinical studies have established the potential efficacy and low toxicity of MCT both in adult and in childhood cancer patients mainly relying on the anti-angiogenic nature of MCT. It was becoming clear at the time that not a single metronomic regimen would fit all tumour types and settings.

Metronomics 2.0 was started when the notion of the multitargeted nature of MCT was acknowledged. MCT was indeed reported to be a multitargeted cancer therapy rather than a simple anti-angiogenic therapy as in addition to inhibiting tumour angiogenesis, MCT can also restore anticancer immune response [8, 9], tilting the immune system from immunosuppression to immunostimulation [10], and inducing tumour dormancy. For instance, it was demonstrated that 5-fluorouracil selectively killed myeloid-derived suppressor cells, strengthening the immune response against the tumour [11]. Also, MCT with cyclophosphamide [12, 13] or gemcitabine [14] or combination of agents [15] modulated the antitumour immune response.

The field of metronomics is moving forward in two seemingly opposite directions. In low- and middle-income countries, the prognosis of patients with cancer remains dismal, because of late diagnosis and incorrect cultural beliefs about cancer and anticancer treatments. The characteristics of metronomics make it a good candidate to meet unmet needs and to treat patients. Indeed, for a majority of patients around the world, anticancer treatments remain too expensive, too toxic, and require well-established and sophisticated health care infrastructures [6].

In high-income countries, metronomics is going ‘personalised’ through its combination with targeted therapies and recently immune check-point inhibitor [16]. And it is at this point, at the end of the timeline, that Metronomics 3.0 can be positioned. The notion that neoadjuvant antiangiogenic therapy can have different effects on primary and metastatic tumours has emerged [17]. Also, it has become clear that the combination of modern therapies as drug repositioning, targeted therapies, and immunotherapies can be used [18], as well as computational metronomics that contributes to optimising the schedule of drug administration [19].

In the last part of his talk, Dr André outlined the launch of the Metronomic Global Health Initiative in order to promote the use of metronomics for children and adults with cancer living in countries with limited resources (metronomics.newethicalbusiness.org). They built a network of individuals, physicians, scientists, and institutions willing to use this strategy for patients. Scientific meetings, international preclinical collaborations, and clinical trials have been initiated worldwide over recent years. Thus, they recently reported, through a Franco-Australian-Indian collaboration, the value of a combination of propranolol and MCT for patients with angiosarcoma [20, 21]. This approach has been adopted by the French sarcoma group that is launching a state-of-the-art phase-I/phase-II trial (NCT02732678) highlighting the potential of reverse innovation, that is the flow of ideas from lower to higher income settings. Metronomics is a strategy that allows LMIC to generate their own treatment and integrate innovation, in a way that is adapted to their local constraints to try to fulfil unmet needs beyond level A evidence [22]. Nevertheless, clinical trials are mandatory to demonstrate how they can help people with cancer worldwide.

## Session 1: Pre-clinic experience

Viviana Rozados from the Institute of Experimental Genetics, School of Medical Sciences, National University of Rosario, Rosario, Argentina, described her *in vitro* and *in vivo* experimental work, looking for cancer treatments with low toxicity. Working with the repositioned drug lovastatin, in lymphoma and sarcoma tumour models, its antimetastatic effect was demonstrated, which was caused, at least, by the decrease in adhesiveness of tumour cells [23] and the inhibition of p21^ras^-signalling pathway [24]. Also, it was shown that the combined treatments with lovastatin + doxorubicin can radiosensitise cancer cells by increasing apoptosis [25]. Moreover, it can reduce *in vivo* rat lymphoma and mouse mammary adenocarcinoma growth [26]. Interestingly, no toxicity was demonstrated along the treatments.

She commented that while looking for another antimetastatic, non-toxic, treatment they studied the effect of the administration of a single-low dose of cyclophosphamide (Cy). The interesting results obtained showed that, in spite of not having an effect on primary tumour growth, it did cause a significant decrease in metastasis development, which was mainly due to modulation of the immune response with decrease in interleukin-10 (IL-10), IL-10R, T-regulatory cell population, TH2/TH1 ratio [27], and Galectin-1 expression [28]. The next step was the study of the therapeutic efficacy of MCT with Cy in lymphoma and sarcoma tumour models. The metronomic regimen induced the permanent regression of 100% of the lymphomas and 83% of the sarcomas. The treatment was devoid of general, hepatic, cardiac, and hematologic toxicity [29]. The treatment caused not only a significant decrease in vascular endothelial growth factor (VEGF) serum concentration, in concordance with the known antiangiogenic effect of MCT, but also an immunomodulatory outcome demonstrated both by the decrease in IL-10 serum concentration and by the fact that there were the absence of tumour regressions, when the same treatment was administered to immune-deficient lymphoma-bearing mice [13].

Leandro Mainetti (Institute of Experimental Genetics, School of Medical Sciences, National University of Rosario, Rosario, Argentina) gave a talk about the therapeutic efficacy of metronomic Cy combined with celecoxib (Cel) or doxorubicin (Dox) in two murine mammary adenocarcinoma models. Mice received Cy in the drinking water, 30 mg/kg body weight/day, Cel 30 mg/kg p.o five times/week or Dox 0.5 mg/kg body weight i.p three times/week as mono treatments or the combination of Cy + Cel or Cy + Dox. The same dosage was used either in the antitumoural or in the antimetastatic model.

He found that MCT with Cy plus Cel-inhibited tumour growth and increased the median survival time, the combined treatment being more effective than each monotherapy; importantly, the treatment significantly decreased the number of metastatic lung nodules and their size, indicating the impairment of both, metastases growth and lung-seeding capacity in each tumour model. Moreover, no toxicity was detected at the hepatic, cardiac, renal, and haematological level [30]. On the other hand, the combination of Cy plus Dox inhibited tumour growth, decreased lung metastases, and increased the median survival time, while having low toxic effect. Again, combined MCT was more effective than each monotherapy [31].

The antitumour and antimetastatic effects achieved with the combination of a chemotherapeutic drug (Cy) plus a repositioned drug (Cel) or the combination of two of the most common chemotherapeutic drugs used for breast cancer treatment (Cy and Dox) may be due to several mechanisms, namely inhibition of angiogenesis through decrease in VEGF serum concentration, inhibition of the life cell cycle by the diminution of tumour proliferation rate, stimulation of the death cell cycle achieved by increase in tumour apoptosis and modulation of the immune response evinced using tumour xenografts in nude mice.

The therapeutic benefits of combined MCT with cyclophosphamide plus celecoxib on mammary adenocarcinomas together with its very low toxicity profile encouraged its translation to the clinic resulting in a phase-II clinical trial for treating advanced breast cancer patients [32].

Mauricio Menacho Márquez (Institute of Experimental Genetics, School of Medical Sciences, National University of Rosario, Rosario, Argentina) gave an overview of the results related to the use of a combination of repurposed drugs for breast cancer treatment. During his presentation, it was mentioned that although discovery of new drugs for cancer treatment is one of the great challenges of modern medicine, this research area is commonly linked to the formulation of new molecules, an expensive and time-consuming process. As an alternative to this issue, he proposed the use of a combination of two drugs in repositioning, metformin (Met), and propranolol (Prop), to treat triple-negative breast cancer, normally the type of breast cancer associated with the worst prognosis and with more limited options for treatment.

He began with the presentation of data for *in vitro* exploration of the effect of this drug combination on proliferation and apoptosis of a panel of triple-negative breast cancer cell lines. Interestingly, he showed that metformin and propranolol worked synergistically inhibiting growth and that this combination was also able to affect cellular events related to metastasis, such as migration or invasion. Also, as a conclusion from different *in vitro* approaches that were shown during the presentation, he explained that probably the main mechanism of action for Met/Prop combination is related to a strong inhibition of mitochondrial bioenergetics and a drastic increase in glycolysis. Then, he presented the *in vivo* results of two different tumour models, showing a potential benefit on using this drug combination to prevent tumour growth and metastasis development. Finally, he concluded the presentation proposing the use of Met/Prop combination as a possible adjuvant treatment for triple-negative breast cancer treatment.

Maria José Rico (Institute of Experimental Genetics, School of Medical Sciences, National University of Rosario, Rosario, Argentina) gave a talk on the comparison between four different combined MCT schemes for mammary tumours; (A) Cy + Cel [30], (B) Cy + Dox [31], (C) Cy + Met, and (D) Met + Prop. All the drugs were administered orally except for Dox. The M-406 triple-negative murine mammary adenocarcinoma was utilised and inoculated in CBi mice. Initially, she presented the results of the latest drug combination studied; the administration of Cy + Met showed antitumour and antimetastatic effects, prolonged survival, while having low toxicity.

In the second part of the talk, she compared the four treatments in order to identify, in the M-406 tumour model, the most effective one regarding antitumour effect, antimetastatic effect, survival, and toxicity. The per cent of reduction of tumour volume with respect to the control group without treatment was different among treatments being higher for treatment B>C>A>D. The per cent of reduction of the total lung metastatic volume was higher for A>B>C>D. Also, the difference in the per cent of increase in the survival time was significant, being higher for B>C>A>D. None of the four drug combinations showed toxicity, measured through the evolution of the body weight and the animal’s characteristic such as motor activity, fur quality, food intake, response to stimuli, conduct, and breathing. The A, B, and C schemes inhibited tumour growth in a similar manner, being smaller the effect for D. The same behaviour was observed related to the antimetastatic effect. Besides, the B and C treatments were the ones that produced the higher effect on survival.

She concluded that taking into account the results of the comparisons the Cy + Dox and Cy + Met schemes were the most effective, having the second combination the advantage of its exclusive oral administration, thus covering fundamental aspects in the oncologic treatment as therapeutic efficacy, better quality of life, along with the always sought low toxicity.

Finally, she posed several questions that the precedent analysis brought about the following: will the genetic background of the tumour bearer and the genetic characteristics of the tumour have an impact on the therapeutic efficacy of a given combination of drugs? [33]. Will the addition of a third drug to the combination improve its efficacy while maintaining its low toxicity? Will it be possible to develop a test for personalised identification of the most effective combinations of drugs?

Paula Schaiquevich (Hospital de Pediatría JP Garrahan, Buenos Aires, Argentina) gave a comprehensive overview on the current treatments of patients with retinoblastoma [34]. She talked about the most commonly used chemotherapeutic agents in the clinics and that current pharmacological treatment of retinoblastoma that relies on high doses of chemotherapy. However, she discussed previous studies in children with solid tumours that showed that MCT provided an alternative to maximum tolerated for better tumour control and a lower rate of adverse events [2]. Specifically, she commented about the experience of MCT on retinoblastoma (Metro-Mali 01 and 02 treatment regimens) and the need for the improvement of tumour growth control in both intraocular and extraocular disease [35, 36]. She also introduced the audience to the concept of pharmacokinetically guided dosing of topotecan (TPT) in paediatric patients with solid tumours subjected to protracted treatment with TPT [37].

Also, she highlighted the importance of pharmacokinetics as a surrogate for drug exposure and efficacy/safety. Despite the fact that several pharmacokinetic and pharmacological studies have been carried out, little is known about the influence of the scheme of treatment on the efficacy in patients with retinoblastoma. In addition, melphalan (Mel) has been introduced in the clinics of retinoblastoma showing a potent antitumour response despite little information is available about the influence of treatment schedule on the efficacy [38]. As metronomic use of Cy improves antitumour response in different solid tumours, she reasoned that a similar schedule could enhance the activity of Mel, also a nitrogen mustard-alkylating agent [39]. Then, she showed the results obtained in her laboratory (and in collaboration with Dr Negrotto, Academia Nacional de Medicina and Dr Carcaboso, Hospital Sant Joan de Deu, Spain) about the sensitivity of commercial and patient-derived retinoblastoma cells and endothelial cell types to a single-weekly dose of TPT or Mel and compared with that obtained after continuous low dosing. A significant decrease in the IC50 was observed following MCT treatment in retinoblastoma and endothelial cell types compared with high single-dose treatment. Metronomic TPT or Mel significantly inhibited *in vitro* tube formation compared to cells treated with a short-term treatment. Interestingly, she showed that none of the dosing modalities induced multidrug resistance mechanism assessed by the expression of *ABC*-transporters. Altogether, MCT may be a valid option for retinoblastoma treatment for treating slowly dividing cells from vitreous seeds or as maintenance therapy after complete remission was achieved with intensive chemotherapy. Metronomic treatment would allow reducing the daily dose and the discussed results should be further studied *in vivo*.

## Session 2: Clinic experience in paediatric cancer

Dr Milena Villarroel (Hospital Luis Calvo Mackenna, National Programme of Paediatric Cancer, Santiago, Chile) talked about the background of the Latin American Cooperative Group (GALOP) Protocol for Ewing Sarcoma (ES) treatment. It was the first multicentre Latin American Protocol that was designed to determine the efficacy and safety of an adapted regimen with combined modality therapy that incorporated 14 cycles of alternate vincristine, doxorubicin, cyclophosphamide/ifosfamide–etoposide) every 14 days (interval-compressed) as shown by the AEWS0031 study [40]. Additionally, the feasibility and toxicity of administering low dose, antiangiogenic chemotherapy (MCT) with vinblastine (VBL) (3 mg/m^2^/dose, weekly i.v.) and Cy (25 mg/m^2^/day, continuously p.o), after intensive chemotherapy, was tested. All metastatic patients received MCT for 54 weeks, while patients with localised disease were randomised (1:1) to receive or not MCT according to the following risk factor: age below versus more than 14-years old, pelvic versus non-pelvic primary tumour, tumour size less versus more than 8 cm and male versus female. Surgical and/or radiotherapeutic local control therapy was given according to tumour site and clinical response [41].

She mentioned that in the early 2000s, a regimen of frequent dosing of cyclophosphamide or vinblastine was termed antiangiogenic scheduling, as angiogenesis inhibition was shown to be responsible for the observed antitumour effect. Different mechanisms of action were postulated, from normalisation of tumour vasculature, the prevention of rapid tumour cell repopulation following chemotherapy, to potentiation of the antivascular activity of chemotherapy.

The potential role of angiogenesis in ES was analysed, noting that EWS-FLI1 may function as a promoter for vascular endothelial growth factor, especially in sarcomas, that are highly vascular tumours [42]. She mentioned the literature background for the use of cyclophosphamide-based MCT, and some of the weaknesses of this therapy, related to the absence of validated biomarkers and randomised phase-II trials [39].

Dr Villarroel referred to the Pilot study of low-dose antiangiogenic chemotherapy with vinblastine/celecoxib MCT along with standard ES treatment multiagent chemotherapy, for patients with newly diagnosed metastatic ES family of tumours, which was a Children’s Oncology Group (COG) phase-II study. The protocol, according to their definitions, was feasible as antiangiogenic therapy, could be delivered for at least 75% of days to 25 or more eligible patients. However, high toxicity in irradiated areas was noted, an effect that limited the usefulness of this protocol. The 24-month EFS for those patients with isolated pulmonary metastases was better than historical controls, although the number of patients was small, the follow up was short, and it lacked contemporaneous controls.

Dr Lauro Gregianin (Department of Paediatrics, School of Medicine, Federal University of Rio Grande do Sul, Porto Alegre, RS, Brazil) presented preliminary data on localised Ewing Sarcoma GALOP Protocol, a multicentric Latin American protocol.

First, he mentioned that, as Dr Villarroel had explained previously, the protocol was designed to determine the efficacy and safety of an adapted regimen with combined modality therapy that incorporated 14 cycles of interval-compressed VDC/IE every 14 days, and the feasibility and toxicity of low dose, antiangiogenic chemotherapy (MCT) with VBL (3 mg/m^2^/dose weekly i.v.) and Cy (25 mg/m^2^/day, continuously p.o), after intensive chemotherapy, patients with localised disease were randomised (1:1) to receive or not MCT during 54 weeks, according to the following risk factor: age below versus more than 14 years old, pelvic versus non-pelvic primary tumour, tumour size less versus more than 8 cm, and male versus female.

He reported that from January 2011 to May 2016, 163 (55 %) out of 306 patients enrolled in the protocol had localised disease. Among this, 71 patients (45%) were randomised to the continuous treatment arm with MCT, 77 patients to the non-metronomic arm, and 15 patients were at pre-randomisation time point. He mentioned that 37/71 patients of the MCT arm had complete data for analysis.

He described data from 216 cycles analysed (1 cycle = 4 weeks of treatment), in which the median dose of Cy/VBL was 22.6 (range 0–37) and 4.03 (range 0–6) mg/m^2^, respectively. He reported that the grade 3–4 haematological toxicities were: anaemia in 4.1%, neutropenia in 51% (grade 4: 10%) thrombocytopenia in 10% of cycles, febrile neutropenia in 7% of cycles, and the only grade 3–4 non-haematological toxicity was hepatic, in 3% of cycles. No toxic death was observed.

Finally, he reported that, in the mean follow-up time of 51 months, the 3-year overall and event free survival (OS, EFS) for all patients with localised disease, irrespective of receiving or not MCT, was 0.891 and 0.686, respectively.

He mentioned that although patients receiving metronomic MCT had an 3-year OS and EFS higher than that observed in patients who did not receive continuous MCT (3-year OS 0.902 vs. 0.795, *p*=0.802, EFS 0.782 vs. 0.652, *p*=0.344), those differences were not statistically significant.

He concluded that preliminary data showed that metronomic MCT with Cy/VBL was feasible, with tolerable toxicity, and additionally, even if it was observed no difference in survival between patients with localised ES receiving or not MCT, an extended follow-up time is needed to draw firm conclusions on the impact of continuous MCT.

Dr Adriana Rosé (Solid Tumour Area – Paediatric Haematology and Oncology Department, Garrahan Hospital, Buenos Aires, Argentina) presented preliminary data of metastatic Ewing Sarcoma GALOP Protocol, a multicentric Latin American protocol with 27 centres from Brazil, Chile, Argentina, and Uruguay.

First, she mentioned that, as Dr Villarroel had explained previously, the protocol was designed to determine the efficacy and safety of an adapted regimen with combined modality therapy that incorporated 14 cycles of interval-compressed VDC/IE every 14 days, and the feasibility and toxicity of low dose, antiangiogenic chemotherapy (MCT) with VBL (3 mg/m^2^/dose weekly i.v.) and Cy (25 mg/m^2^/day, continuously p.o), after intensive chemotherapy, for all metastatic patients, during 54 weeks.

She reported that from January 2011 to May 2016, 138 out of 306 patients (45%) enrolled in the Protocol had metastatic disease. She highlighted the high incidence rates of metastatic disease compared with other series. The median age reported at diagnosis was 12.5 years (1–26), most were male (M/F rate was 2.3/1), with axial primary site in 60% of patients. The response rate to induction therapy was 77%.

She mentioned that for metastatic ES, 54 patients (39 %) received MCT as planned; among this, 40 patients were evaluable for analysis, 22 patients (16 %) were on protocol (VAC/IE), and 62 patients (45%) did not received MCT as planned, mainly due to progressive disease (32 patients).

For 356 analysed cycles of MCT (4 weeks per cycle) among 40 patients, median number of cycles per patient was 8.9 (1–27), and the median Cy and VBL dose was 22.2 mg (12.2–2.8), and 3.5 (2.96–3.91), respectively.

She reported that grade 3–4 haematological toxicities were: anaemia in 18 (5, 1%) cycles, neutropenia in 95 (27%), thrombocytopenia in 4 (1%), and non-haematological toxicity were as follows: febrile neutropenia in 4 (1%), elevation of creatinine in 1 cycle. No grade 3–4 toxicity was reported for mucositis, diarrhoea, hepatic or weight loss.

Finally, she presented preliminary data on survival, and mentioned that, with a median follow-up of 33 months (3–55), the 3-year p0S was 0.59 versus 0.23, and 3-year pEFS 0.51 versus 0.23 for metastatic patients with or without MCT, respectively.

She concluded that MCT with Cy/VBL was feasible and safe, and although it could be affected by bias because metastatic patients who did MCT were good responders to intensive chemotherapy; the OS and EFS of the metastatic patients with MCT was better than without MCT; however, an extended follow-up is needed to draw firm conclusions.

Jaume Mora gave an overview of the Spanish multi-institutional experience with Ewing Sarcoma (ES) treated in a phase-II study using intensive chemotherapy including gemcitabine/docetaxel (G/D) as maintenance treatment for children and adults.

He initially described the immunohistochemistry, molecular features and evolution of chemotherapy in ES. The first part of his talk was related to the background of the GEIS – 21 trial, based on two previous protocols: 1 – the modified P6 (mP6) that consisted of three courses of CDV (cyclophosphamide/vincristine/doxorubicin) and IE (ifosfamide 14 g/m^2^/course, instead of 9 g/m^2^ for poor histological responders, and etoposide), with a 4-year OS and EFS of 92% versus 42%, and 83% versus 28%, for localised versus metastatic disease, respectively [43]; and 2 – the pilot study of G/D for advanced paediatric sarcomas with a reported overall response rate (ORR) of 63%, without major toxicity [44]. He also mentioned the preclinical experience that showed G/D as an active regimen in relapse/refractory ES xenograft models, and the heterogeneity of response among ES [45].

In the second part, he talked about the GEIS-21 Trial describing the guidelines of the protocol. The aims of the study were to reproduce the mP6 results and to test the efficacy of G/D in newly diagnosed, previously untreated, high-risk (HR) ES patients, in a prospective, multicentric, non-randomised study, including patients ≤40 years with ES and proved rearrangements of *EWSR1*. HR patients were those with pelvic or axial primary tumours, and/or metastasis, and/or BM micrometastasis. All patients received five mP6 courses; surgical resection after course 3; and radiotherapy after course 5. High-risk (HR) patients received two window cycles of G/D (G at 1.000 mg/m^2^ iv on days 1 and 8, and D at 100 mg/m^2^ on day 8 of a 21-day cycle); HR patients with an OR to G/D, received maintenance with 12 monthly/cycles of G/D after mP6. The trial was opened between April 2010 and December 2014.

Finally, he reported the results of the 43 patients enrolled in the trial, median age was 17 y (range 3–40), 22 patients (51%) were standard risk (SR) and 21 patients HR. Among 10 of 21 HR patients evaluable for G/D window therapy response, the ORR was 70%. At the end of mP6 30, out of 43 patients were evaluable, and the ORR was 93% (7% progressive disease); 11/21 HR entered to G/D maintenance and 1 year later 50% remain in CR. The statistically significant differences were detected for risk groups: SR 4-y OS 74% (CI=51,100) and 4-y EFS 67% (CI=47,95); HR 4-y OS 42% (CI=25,73) p=0.011 and EFS 27% (CI=13,56) p=0.0028; and age: <18y 4-y OS 78% (CI=58,100) and 32% for >18y (CI= 15,69) p<0.001; 4y-EFS 62% (CI= 43,89) <18y and 28% (CI= 13,61) >18y, p=0.0087. Cox model for HR and >18y versus SR and <18y, p<0.001; 18y versus same risk group, p= 0.0021, superior to same age versus risk group, p=0.014. His conclusions of the trial were that G/D regimen resulted in objective responses in 70% HR-ES, age was stronger predictive marker than risk group, and monthly G/D benefited 50% of HR-ES providing a backbone regimen for managing minimal residual disease.

Wendy Gómez García from the Onco-Haematology Department of Dr Robert Reid Cabral Children’s Hospital, Dominican Republic, gave a comprehensive overview of the experience on MCT in a Palliative Care Programme in a Low Income Country.

Initially Dr Gómez talked about MCT, its definition and its antiangiogenic and immunomodulatory effects [46, 47] and the metronomic approaches in Paediatric Oncology [6, 48]. Then, she presented the experience in Dominican Republic, member of AHOPCA (Centro American Paediatric Haemato-Oncology Association) from January 2012 to January 2016. Fifty-seven patients (16%) among 357 new cases of cancer were admitted to the Palliative Care Programme & Metronomic Therapy (PCP & MT) due to refractory disease, metastatic disease at diagnosis with high-risk psychosocial factors, and/or progressive or relapsed disease after second or third line of treatment.

Among this group, they were between 5 and 9 years old, of which 55% were male. The therapeutic option chosen by the parents was the comprehensive support with metronomic therapy (MCT) in 77% of cases. Among this group, 41% had more than 24 weeks half-life, while 100% of patients who were treated only with comprehensive support without MCT survived less than 12 weeks. The AHOPCA MT Protocol consist of the combination of Ibuprofen 20 mg/kg/day for 42 days, Cy 25 mg/m^2^/day for day 1–21, methotrexate 7.5 mg/m^2^/day twice a week from day 21–42, DR use 6-mercaptopurine 25 mg/m^2^/daily because of the lack of Cy. The use of opioids in Palliative Care began with the PCP & MT in 2012, and it is, presently of 56%.

She highlighted the importance of promoting the comprehensive support and palliative care of patients with cancer, mainly in those who do not have curative treatment, with the aim of contributing to maintain stable disease, to relieve pain and discomfort, to improv*e* functional and social activities, to achieve a better quality of life, and to give peace of mind to the families that their son or daughter are receiving adequate medical attention and medication.

In summary, she considered that MCT is cheap and easily available, uses oral drugs in an ambulatory setting, with low incidence of side-effects, feasible in a low-income Country, for the treatment of advanced disease with the aim of maintaining stable disease. She also said that more research is necessary to provide more evidence-based results (i.e., biomarkers), with a larger number of patients and the use of standardised tools for measuring quality of life [49].

She closed her talk with the statement that ‘Never say: There is nothing to do. Usually when you think on this phrase is when more things you can offer….Metronomic Therapy is one of them’.

Dr Epelman from the Paediatric Oncology Department, Santa Marcelina Hospital, São Paulo, Brazil, first mentioned the need to improve cancer care in low- and middle-income countries (LMIC), with the development of comprehensive care centres for paediatric oncology [50].

He went on to comment that the advances in paediatric brain tumours (PBT) have been less successful than in other areas of paediatric oncology, essentially related to specific aspects of these tumours in this age group, such as the fact that the surrounding brain is still developing, vital structures limit resection, drug penetration into the CNS is often poor, and short- and long-term toxicities are significant, so new therapeutic approaches in paediatric neuro-oncology are needed.

He mentioned that although the number of new active antineoplastic agents has been scarce during the last decade, significant improvements in the chemotherapeutic management of PBT have been observed, related to the optimisation of schedules, sequential high-dose chemotherapy, concomitant administration of chemotherapy and radiation, or the introduction of intrathecal or intraventricular chemotherapy in specific protocols.

He also referred to the role of molecular biology and its impact in diagnosis, prognosis, and the treatment of PBT, and to the necessity of identifying biologic pathways and to develop a risk stratification based on clinical and biologic prognostic factors [51, 52].

Regarding the use of metronomic dosing for PBT, he said that this therapy had demonstrated some encouraging results, using single agents such as topotecan [53] or etoposide, in multiple brain tumours, with response rate from 50 to 80%, and with multi-agent regimens such as bevacizumab, thalidomide, celecoxib, fenofibrate, etoposide and cyclophosphamide, COMBAT (temozolomide, etoposide, celecoxib, vitamin D, fenofibrate, retinoic acid), or thalidomide, celecoxib, low dose etoposide and cyclophosphamide, which have demonstrated some benefit in patients with relapsed PBT [54], or bevacizumab and irinotecan followed by metronomic maintenance with weekly vinblastine, in low-grade gliomas [55].

The last part of his talk was about the use of MCT for recurrent or refractory PBT and the role of antiangiogenic therapy as an alternative modality with limited acquired resistance that may offer some benefit to patients treated initially with intensive regimens. He mentioned that the propensity of malignant tumours to develop drug resistance is one of the major problems of cytotoxic strategies and that antiangiogenic therapy is an option, since all tissue growth is angiogenesis dependent and the angiogenic stability of host endothelial and vascular-progenitor cells targeted by antiangiogenic therapy should have limited acquired resistance However, dosing and duration of such palliative treatment have not been systematically studied, and there is also uncertainty of when to stop MCT. He also posed questions about the best time to use MCT: during treatment, in the relapsed setting during upfront treatment, or as maintenance? Long-term toxicities must be well evaluated to avoid an increased risk of second malignancies, mainly when using alkylating agents like cyclophosphamide, etoposide, or temozolamide.

He concluded that early results with MCT are promising and can be considered as a therapeutic option for LMIC, pointing out that further investigation is warranted to determine which agents show better benefit in PBT and underlying the need to incorporate novel biological markers into current protocols.

## Session 3: Clinical experience in adult cancer

Carlos Alasino (Institute of Oncology of Rosario, Rosario, Argentina) highlighted the results of the metronomic administration of Cy and Cel in the treatment of metastatic breast cancer patients. This combination only showed grade-I and grade-II haematological and gastric toxicities in a low percentage of patients. The main clinical outcomes were prolonged stable disease (PSD>24 weeks) in 10/20 patients (50%) and partial remission (PR) in 1/20 (5%). No complete remissions (CR) were observed. The clinical benefit was high for this kind of patient, reaching 55% [32]. Interestingly, the overall survival was significantly higher in patients with clinical benefit.

He then showed the results obtained analysing several putative biomarkers of response such as circulating endothelial cells (CEC), circulating progenitor endothelial cells (CEP), serum concentration of VEGF, VEGF-C, soluble VEGF receptors 2 and 3 (sVEGFR-2, sVEGFR-3) and TSP-1. Apart from confirming that the drug combination was antiangiogenic, some relevant findings indicated that increased levels of CEC could be useful for detecting progression and, more importantly, the baseline levels of VEGF or the VEGF/sVEGFR-2 ratio could be useful as early predictors of response because they were significantly correlated with TTP [51].

Another subject that he discussed was their thorough studies on the quality of life (QoL) of the patients during treatment, an assessment carried out by FACT-B questionnaire, Brief Pain Inventory and ECOG scale. After the metronomic treatment of several months, a high proportion of patients showed improvement or no changes in their QoL, and there were fewer patients with pain at the end of the treatment [52].

Daniel Alonso (Laboratory of Molecular Oncology, National University of Quilmes, Buenos Aires, Argentina) gave an overview of his experience in the repurposing use of desmopressin (dDAVP), a synthetic peptide analogue of the antidiuretic hormone originally described in the late sixties, in surgical oncology. The compound is a selective agonist for the V2 vasopressin membrane receptor, mediating antidiuretic, and haemostatic responses in kidney-collecting ducts and endothelial cells, respectively [53]. The presence of vasopressin receptors has been also documented in various human malignancies, including breast, colorectal, and small-cell neuroendocrine cancer [54].

His group had reported for the first time that dDAVP was capable of inhibiting lung colonisation by bloodborne tumour cells in preclinical mouse models of aggressive breast cancer [55, 56]. Besides, perioperative administration of dDAVP significantly prolonged survival in a clinical veterinary trial in dogs with locally advanced mammary cancer [57]. Alonso summarised evidence on mechanisms of action that account for the antitumour activity of dDAVP, including a direct cytostatic effect, stimulation of microenvironmental production of angiostatin and endothelial release of von Willebrand factor, a key element in resistance to metastasis. He suggested that dDAVP breaks cooperative interactions of tumour and endothelial cells during early metastatic progression.

A recently finished phase-II dose escalation trial in breast carcinoma patients explored safety and potential utility of perioperative administration of dDAVP in humans (NCT01606072). At the highest dose level evaluated (2 μg/kg) dDAVP appeared safe when administered in two slow infusions, before and after surgery. Interestingly, treatment with dDVAP was associated with a reduced intraoperative bleeding and a rapid postoperative drop in circulating tumour cells, as measured by quantitative PCR of cytokeratin-19 transcripts [58]. A trial in patients with rectal bleeding due to colorectal cancer is ongoing (NCT01623206). He concluded saying that perioperative period is an attractive window of opportunity to reduce the risk of metastatic disease. In this context, dDAVP emerges as a potential surgical adjuvant in oncology.

## Summary and perspectives

Guillermo Chantada (Hospital JP Garrahan, Buenos Aires, Argentina) made a precise synthesis of the preceding talks underlying the excellence of local and regional developments and concluding that it was evident that the use of MCT is an established practice in Latin America. He highlighted several aspects of the presentations such as the experiences in preclinical models of mammary tumours, which derived from associations for developing projects between a research centre and two hospitals, the ongoing multicentric protocols with randomised branches in metronomics, the *in vitro* studies in retinoblastoma and the drug repositioning in mammary and retinoblastoma tumours. He mentioned the importance of the protocols in osteosarcoma and the approved one of immunotherapy and metronomics in neuroblastoma and also the repurposed use of vasopressin in a perioperative administration. He continued by making a summary of the preclinical evidence shown in the meeting related to the *in vitro* and *in vivo* activity for paediatric and adult tumours, the multiple mechanism of action implied in the use of propranolol for angiosarcoma, the effects on cellular toxicity, immunity, vasculature and microenvironment and talked about reverse innovation. Then, he called attention on the limitations in the *in vitro* studies like drug delivery in 3D tumours or the absence of cellular heterogeneity and in the *in vivo* models with tumour and without immune system or vice versa, also pointing at the lack of biomarkers of response to the treatment.

From the paediatric perspective, he referred to the use of MCT mostly in palliative care as is the case in Central America, a setting where many patients with solid tumours present advanced incurable disease in a context of poor resources and socioeconomic problems limiting the possibility of using conventional chemotherapy. In addition, metronomic chemotherapy is currently incorporated in first line therapy of high-risk bone tumours in the context of multicentric international studies in the region through the GALOP. Moreover, he remarked that a new perspective of association of immunotherapy with an anti-idiotype vaccine and metronomic chemotherapy, targeting minimally disseminated disease, has just gained regulatory approval in Argentina as a phase-II study of which preliminary results were shown about toxicity and final results on patient survival are still pending.

Importantly, he stated that the major challenge for the use of MCT in retinoblastoma and CNS tumours would be to concomitantly develop innovative tools for drug delivery to these sites that are difficult to reach from the systemic route. More data on the pharmacokinetics of MCT in different paediatric malignancies are needed, as well as adequately designed clinical trials to address its role in tumours with different biology.

In conclusion, G Chantada ended by saying that there are strong groups working on MCT in Latin America, both in the preclinical and the clinical settings, including large, adequately designed randomised studies that will show relevant data for the scientific community. The potential use of MCT, as a cost-effective treatment in low resource clinical setting may be considered also for adjuvant therapy in selected tumours. The role of the governmental agencies and non-governmental alliances as the Metronomic Global Health Initiative, in supporting this research with public interest should be important, since funding from the pharmaceutical industry for these initiatives would be unlikely.
